# Composition and Bioactivity of Alentejo *Calamintha nepeta* Essential Oil: The Impact of Seasonality and Climatic Stress on Antioxidant Capacity and MDR Antibacterial Potential

**DOI:** 10.3390/molecules31122100

**Published:** 2026-06-15

**Authors:** Sílvia Macedo Arantes, Andreia Piçarra, A. Teresa Caldeira, M. Rosario Martins

**Affiliations:** 1 HERCULES Laboratory—Cultural Heritage Studies and Safeguard & IN2PAST Associate Laboratory for Research and Innovation in Heritage, Arts, Sustainability and Territory, Institute for Research and Advanced Training (IIFA), University of Evora, 7004-516 Evora, Portugal; saa@uevora.pt (S.M.A.); andreiapicarra@hotmail.com (A.P.); atc@uevora.pt (A.T.C.); 2 HERCULES Laboratory—Cultural Heritage Studies and Safeguard, Institute for Research and Advanced Training (IIFA), University of Evora, 7004-516 Evora, Portugal; 3 Chemistry and Biochemistry Department, School of Sciences and Technology, University of Evora, 7004-516 Evora, Portugal; 4 City U-Macau Chair in Sustainable Heritage & Sino-Portugal Joint Laboratory of Cultural Heritage Conservation Science, Institute for Advanced Studies and Research, University of Evora, 7000-809 Evora, Portugal; 5 Department of Medical and Health Sciences, School of Health and Human Development, University of Evora, 7000-671 Evora, Portugal

**Keywords:** *Calamintha nepeta*, essential oil, seasonal fingerprinting, climatic stress, chemical composition, antioxidant properties, antimicrobial potential

## Abstract

Essential oils (EOs) from wild *Calamintha nepeta* (Portugal) populations collected in Portugal (Évora) were investigated in order to evaluate the impact of Mediterranean seasonal conditions on their phytochemical composition and biological activity. Essential oil GC-FID and GC-MS analyses revealed distinct seasonal chemotypes, with spring samples dominated by isopulegone/pulegone, whereas autumn samples contained higher proportions of isomenthone and menthol. Antioxidant activity was assessed through lipid peroxidation inhibition, DPPH radical scavenging and ferric reducing power assays, while antibacterial activity was evaluated against multidrug-resistant (MDR) clinical isolates. Seasonal differences were reflected in both EO chemical composition and bioactivity. Autumn samples displayed greater antioxidant potential, with Y1A showing the highest inhibition of lipid peroxidation (IC_50_ = 0.85 mg/mL) and Y2A exhibiting the highest ferric reducing power. Conversely, spring samples were more active against MDR bacteria. Among them, Y1S showed the broadest antimicrobial spectrum, with MIC values ranging from 465 to 1767 μg/mL. The unusually wet spring season coincided with marked isopulegone accumulation (≈50%), while warmer autumn conditions favoured higher levels of isomenthone and menthol in the EOs. These findings highlight the importance of seasonal environmental conditions in determining the phytochemical profile and bioactive potential of *C. nepeta* EOs, providing valuable insights for their standardisation and valorisation in pharmaceutical, food and conservation-related applications.

## 1. Introduction

*Calamintha nepeta* (L.) Savi subsp. *nepeta* (syn. *Clinopodium nepeta* (L.) Kuntze and previously classified as *Calamintha baetica* Boiss. & Reuter), commonly known as calamint, is an aromatic perennial herb of the Lamiaceae family, native to the Mediterranean region. This species thrives in harsh or slightly shaded environments, usually along roads and hedgerows, particularly on calcareous soils, and is characterised by its small, oval leaves, lilac flowers, and bush-like growth, reaching up to 80 cm in height [1]. In Portugal, *C. nepeta* is prevalent in several regions, including Alentejo, where it is not only used as a culinary herb but also in traditional medicine as an expectorant, antiseptic, and spasmolytic [2,3]. Its historical use highlights its importance in local diets and ethnopharmacological practices, emphasizing its relevance as a subject of scientific study [3]. Essential oils (EOs) extracted from *C. nepeta* have gained special attention due to their diverse biological activities, including antioxidant, antimicrobial, anti-inflammatory, and sedative properties [2,3,4,5,6,7,8,9,10,11,12,13,14,15,16,17,18,19,20,21,22,23,24,25,26,27,28,29]. In recent years, the pharmacological and industrial interest in *C. nepeta* has intensified, with contemporary screenings reinforcing its multifaceted bioactivity and positioning its volatile extract as a promising, biocompatible matrix for both the functional food sector and targeted therapeutic applications [30,31]. These properties are intrinsically linked to the chemical composition of the oils, which predominantly include oxygenated monoterpenes of the *p*-menthane skeletal type, such as pulegone, menthone, isomenthone, and their biosynthetic precursors, notably isopulegone [2,3,10,18]. Notably, *Calamintha nepeta* EOs presented severe variations in their chemical composition according to geographic features, and different chemotypes have been recognized [2,3,9,10,18,32,33], each with unique dominant compositions and corresponding biological activities. This diversity has profound implications for the commercial and therapeutic applications of the oils, and more research is needed into the conditions that optimize their production and efficacy [2,10,32,33].

Studies on *C. nepeta* essential oils suggest the existence of two main chemotypes: one characterized by the predominance of pulegone, menthone, menthol, and/or their isomers, and another chemotype in which piperitenone oxide and/or piperitone oxide are the main components [12,13,15,17,21,22,25,34]. Studies comparing the chemical profile of *C. nepeta* EOs from Portugal and Italy reported that Portuguese essential oils were dominated by isomenthone, 1,8-cineole, and isopulegone, while the Italian oil had pulegone as its main compound [2]. Studies on *C. nepeta* from southern Portugal have also identified 1,8-cineole, isopulegol, and isopulegone as the dominant components [2,14], highlighting the chemical polymorphism present in *C. nepeta* EOs, with substantial differences based on geographic, environmental, and seasonal variations. This complex chemical variability highlights the need for region-specific studies to improve the production and applications of *C. nepeta* EOs, ensuring their sustainable use in the pharmaceutical, food, and agricultural industries.

Despite the wide recognition of the biological activities of *C. nepeta* EOs [5,6,15,16,20,22,26,27,35], several aspects remain little explored. There is a need for a comprehensive understanding of how seasonal and geographic factors influence the chemical profiles and biological potential of oils. For example, although the antimicrobial potential of *C. nepeta* EOs is well documented [8,15,28,36], there are still questions about the interaction of minor components and their synergistic effects. This study aims to address these gaps by examining seasonal variations in the chemical composition of *C. nepeta* essential oils collected from Portuguese populations. By correlating these variations with the antioxidant and antimicrobial properties of the oils, the research seeks to elucidate the key factors that influence their bioactivity. Crucially, since the Mediterranean basin represents one of the territories most vulnerable to climate fluctuations, understanding the impact of environmental anomalies has become a key priority. Recent insights into Mediterranean Lamiaceae have emphasized that climate change-related abiotic drivers deeply modulate the plants’ secondary metabolism, significantly shifting both the ultimate yields and the specific chemical ratios of major commercial components [37]. The purpose of this research is to evaluate how extreme and atypical climatic events (thermal, hydric, and mechanical stress) influence the volatile profile and biological activities of *C. nepeta.* By providing insights into plant metabolic resilience under a changing climate, this work supports the standardized and sustainable use of these essential oils. Furthermore, it contributes to a deeper understanding of how environmental shifts impact the chemical quality and potential industrial applications of this species.

## 2. Results and Discussion

### 2.1. EOs Characterization

#### 2.1.1. Physical Parameters and Extraction Yields

The physical parameters of *C. nepeta* EOs and their extraction yield are summarized in Table 1. The yields ranged from 0.44% to 0.80% (*v*/*w*), which is consistent with reported values for Lamiaceae plants, which are generally less than 1%. The highest yield was obtained from the Y2A harvest (0.80 ± 0.04%). The observed fluctuations in EO yields (0.44% to 0.80%) can be attributed to the plant’s adaptive response to environmental conditions and its phenological stage [32,38]. The significantly higher yield in Y2A likely reflects a defensive metabolic response to the cumulative thermal stress and lower precipitation recorded during that period. In the Lamiaceae family, increased temperature and solar radiation often stimulate the biosynthesis of essential oils in glandular trichomes as a mechanism to mitigate oxidative stress and water loss. Conversely, the lower yield in Y2S may be related to the intensive vegetative growth phase, where metabolic energy is primarily directed towards biomass production rather than secondary metabolite accumulation [38,39].

Regarding the physical properties, density (0.927–0.989) and refractive index (1.46–1.47) values remained stable across all seasons, without significant differences (*p* < 0.05), serving as primary indicators of the oils’ quality and purity. According to Başer and Buchbauer [38], the refractive index of essential oils, one of the first analytical methods for the systematic study of essential oils, should be between 1.45 and 1.59.

#### 2.1.2. Chemical Composition and Seasonal Polymorphism

The chemical composition of the essential oils, based on relative quantitative data obtained by GC-FID analysis, is presented in Table 2. Regarding the chemical profile of the EOs, it was observed that they are mostly composed of oxygenated monoterpenes, representing 92.9% to 96.1%. A significant seasonal polymorphism was observed: Spring EOs (Y1S and Y2S) were characterized by a higher content of *p*-menthane-type ketones (isopulegone and pulegone up to 49.6% and up to 13.8%, respectively), whereas Autumn EOs (Y1A and Y2A) showed a marked increase in isomenthone (up to 31.9%), and menthol (up to 17.8%). The EOs from both seasons also showed 1,8-cineole (up to 16.3%) as one major component. When comparing the chemical profiles of the spring essential oils Y1S and Y2S, we observed the metabolic stability of the chemical classes of compounds, although the distribution of individual components shifted. Sample Y2S was collected during a spring characterized by an extreme precipitation event (the wettest spring in the last 15 years, with precipitation levels in the Alentejo region showing an increase of more than 164% humidity relative to the historical average [40]), showed an accumulation of isopulegone (49.69 ± 0.04%), in contrast to the chemical profile of Y1S with a more balanced distribution between isopulegone (44.7%) and pulegone (13.8%). This extreme water availability and reduced solar radiation could have inhibited the final steps of the pulegone biosynthetic route.

In the Lamiaceae family, the conversion of isopulegone to pulegone is a key enzymatic step mediated by isopulegone isomerase, and the environmental stressors, such as high precipitation and reduced solar radiation, as observed in Y2S, have been shown to modulate enzymatic efficiency in the p-menthane pathway, leading to the accumulation of the precursor isopulegone [43,44]. Recent studies in Lamiaceae species have increasingly demonstrated that climate change-driven abiotic stresses, such as atypical drought, thermal oscillations, and altered rain patterns, act as critical triggers that reshape secondary metabolic pathways, significantly forcing the accumulation of specific target volatile compounds over others [37,45,46]. Regarding sample Y2A, an increase in isomenthone (31.98 ± 0.24%) and menthol (17.82 ± 0.54%) coincides with exceptional climatic conditions, considered the second warmest autumn in Portugal since 1931, specifically marked by the warmest October in the last 80 years, with record high minimum temperatures [40]. This thermal stress, combined with the mechanical stress of a tornado event in the region, likely accelerated the monoterpene reduction pathway, promoting the conversion of pulegone into its saturated derivatives (isomenthone and menthol isomers), more intensely than observed in the previous autumn (Y1A), as an adaptive strategy to stabilize the chemical profile against oxidative heat stress [10,33,47].

The seasonal and interannual variability of the studied essential oils reveals highly significant differences among the major volatile constituents, specifically within the p-menthane ketone/alcohol pairs pulegone/isopulegone and menthone/isomenthone, driven both by the harvesting season and the specific year of harvest. As statistical analysis confirms, the major volatile constituents of the four *C. nepeta* essential oils displayed distinct and statistically significant differences (*p* ≤ 0.05), marked by the non-overlapping lowercase letters in Table 2. In the spring EOs, the dominance of ketones was validated by the significantly high levels of isopulegone, which peaked in the exceptionally rainy season of Y2S at 49.7%, establishing a clear statistical separation from the 44.7% observed in Y1S. Simultaneously, pulegone values shifted significantly from 13.8% in Y1S to 5.1% under the thermal extremes of Y2A. For the autumn chemotypes, the metabolic redirection towards saturated derivatives was corroborated by the significant accumulation of isomenthone, which reached its maximum in Y2A at 31.9% compared to only 7.4% in Y2S, while menthol also marked a statistically significant maximum in Y2A at 17.8%. Furthermore, the 1,8-cineole exhibited a continuous and statistically significant upward trend across all evaluated periods, rising from 6.9% in Y1S to its absolute peak of 16.30% in Y2A. The observed variability in the chemical composition is consistent with the literature, which identifies two main chemotypes of *C. nepeta* essential oils: the “ketone-type”, characterized by the predominance of pulegone and menthone, menthol and/or their isomers, piperitenone, piperitone, and their oxides [12,13,15,22,25,34], and the “oxide-type”, characterized by the dominance of piperitenone oxide and/or piperitone oxide [17,21,22]. Our samples clearly align with the “ketone-type”, isopulegone/pulegone chemotype typical of the Portuguese flora [2,8].

The chemical variability of *C. nepeta* extends to other regions of the Mediterranean and Central Asia. Marongiu, et al. [2] compared the chemical composition of EOs of Portuguese and Italian *C. nepeta* and reported the presence of isomenthone, 1,8-cineole, and isopulegone as main components of Portuguese EO, while pulegone was the dominant component of Italian EO. Whereas other studies reported the presence of 1,8-cineole, isopulegol, and isopulegone as major components of the EOs of *C. nepeta* from some Mediterranean regions [10]. In Azerbaijan, an atypical chemotype rich in thymol was identified [29], while in Algeria, the major components observed were oxygenated monoterpenes, namely pulegone (58.36%), isoborneol (10.40%), menthone (8.91%), and piperitenone (3.86%) [28]. In Turkey, Alan, et al. [48] found three distinct profiles of essential oils extracted by hydrodistillation: (i) piperitone oxide (44.4%), piperitenone oxide (11.7%) and limonene (7.1%); (ii) pulegone (11.9%), menthone (11.9%), carvacrol (10.0%) and limonene (7.5%); iii) trans-piperitone oxide (30.9%), caryophyllene oxide (7.8%). In Tunisia, Debbabi, et al. [42] reported that the essential oil of *C. nepeta* subsp. *nepeta* was mainly composed of oxygenated monoterpenes (82%), namely piperitone oxide and piperitenone oxide (51.7% and 23.4%). More recently, investigations on Mediterranean populations of *C. nepeta* have further highlighted the extensive plasticity of this species, mapping diverse chemotypes with broad-spectrum antimicrobial and pharmacological potencies tailored by localized geographic and eco-environmental variants [30,49].

It is important to note that the evolution of the plant’s life cycle is accompanied by a distinct biosynthetic drift in its secondary metabolism. As the seasons progress and the end of the vegetative cycle approaches, a decrease in the relative percentage of pulegone is observed, which likely reflects a slowdown in the metabolic pathways responsible for ketone synthesis. In parallel, the increase in the content of monoterpenols (alcoholic monoterpenes such as menthol and its isomers) can be interpreted as an adaptive response to severe water stress and high temperatures, conditions characteristic of late summer in the Alentejo region, a climate classified as Csa [50]. This variability confirms that the chemical fingerprint of *C. nepeta* essential oil is not static, being modulated by the plant’s phenology and local environmental pressures, which favour the accumulation of more stable oxygenated compounds over the majority of ketones typical of the pre-flowering or early flowering stages [10,33,47].

This chemical variability confirms that EO composition is determined not only by genetic factors (genus, species, and subspecies) of aromatic plants, but also by external factors such as geographic location, environmental conditions, and harvest season [33], suggesting that the secondary metabolism of the plant and the production of terpenes are strongly influenced by its life stage and the ecological conditions [33]. In fact, the significant presence of pulegone isomers (such as isopulegone and isopulegol) suggests that the chemical profile may be influenced by physicochemical transformations (hydrolysis and thermal decomposition) that occur during the extraction process [39,51]. These compounds are often considered “hydrodistillation artefacts” rather than primary metabolites present in vivo [38,51]. Although hydrodistillation is the standard method described by the pharmacopoeia for obtaining essential oils, this process subjects the plant material to temperatures close to 100 °C in an aqueous medium for prolonged periods [38]. Hydrodistillation functions as a chemical reactor: under these conditions, oxygenated monoterpenes, particularly α,β-unsaturated ketones such as pulegone, are susceptible to thermal rearrangements and isomerization processes [52]. Beyond thermal factors, the allylic position of the double bond in pulegone makes it highly reactive under the mildly acidic conditions that often develop during the hydrodistillation of Lamiaceae species [53]. In addition, the presence of monoterpenols, such as isopulegol or menthol, may result from the hydrolysis of non-volatile glycoside precursors, which release their terpene aglycones through thermal and hydrolytic processes [52].

Furthermore, in the present study, all samples were subjected to the same conditions for essential oil extraction, and striking differences in the chemical profile of each sample strongly suggest that the chemical variations reflect a primary biological response to climatic stressors, rather than being mere technical artefacts. Examples of this are sample Y2A (isomenthone 31.98 ± 0.24% and menthol 17.82 ± 0.54%), collected after a period of extreme thermal instability and intense mechanical stress (including tornado activity and strong cyclonic winds in the Alentejo region), and sample Y2S, rich in isopulegone (49.69 ± 0.04%), which reflects the metabolic impact of exceptional levels of precipitation and localized convective storms. These events likely triggered thigmomorphogenetic responses, a mechanism known in plants of the Lamiaceae family that redirects the secondary metabolism of these plants towards the production of volatile defence compounds, providing a plausible explanation for the emergence of the unique chemical profiles, rich in isopulegone or isomenthone and menthol, observed in these samples, which distinguish them from the chemical profiles produced under more stable climatic conditions [40].

Thus, the observed chemical profile represents the volatile fingerprint resulting from the interaction between the plant’s secondary metabolism (influenced by the seasonality of Alentejo) and the experimental conditions of the extraction method. This phenomenon is particularly relevant for the standardization of essential oils intended for commercial or industrial uses, since small changes in the proportion of their chemical constituents and the resulting synergistic effects can significantly alter the bioactivity and toxicity of the essential oil [38,54].

### 2.2. Antioxidant Properties of C. nepeta EOs

**Figure 1:**
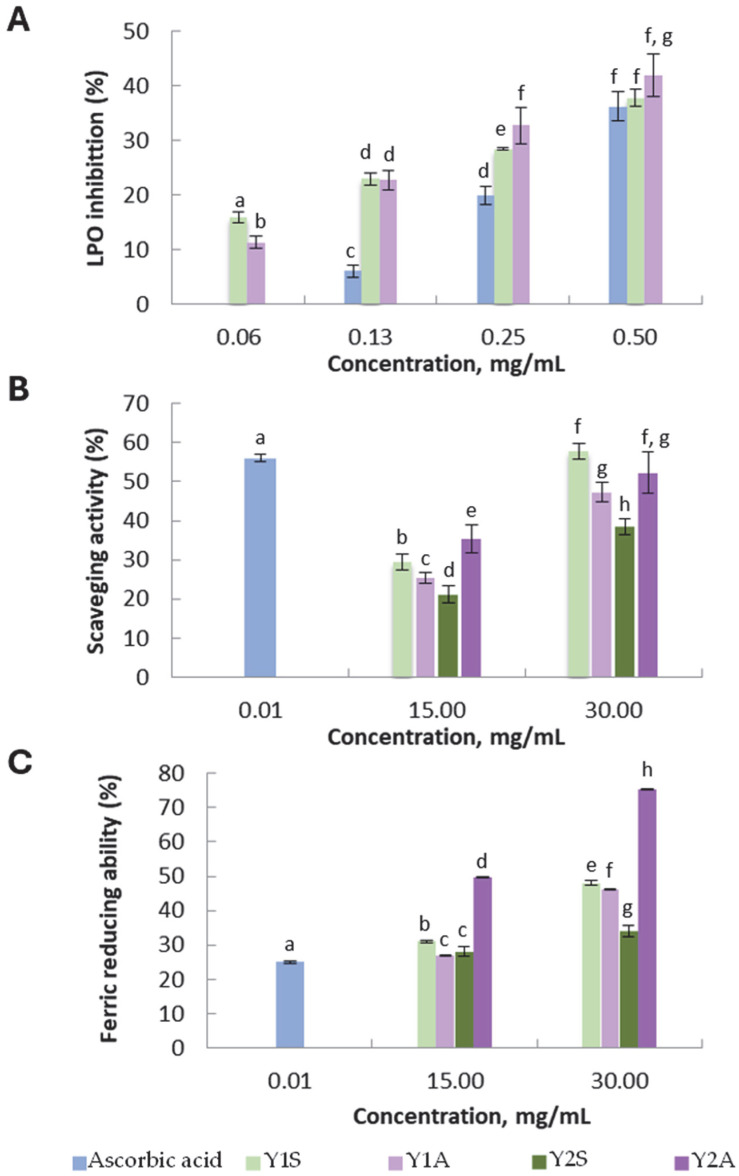
Antioxidant activity of *C. nepeta* essential oils determined by lipid peroxidation inhibition (**A**) DPPH radical scavenging activity; (**B**) and ferric reducing power (**C**). Y1S—Year 1 Spring; Y1A—Year 1 Autumn; Y2S—Year 2 Spring; Y2A—Year 2 Autumn. For graphical comparison, representative concentrations common to all samples were selected for presentation. Bars marked with different letters indicate statistically significant differences among samples and concentrations according to ANOVA and the Tukey test (*p* < 0.05).

Results of antioxidant properties are reported in Figure 1 and Table 3. EOs of *C. nepeta* exhibited high antioxidant activity in the three tested methods, indicating their potential to inhibit lipid peroxidation and act as radical scavengers and ferric reducers.

The antioxidant activity of *C. nepeta* essential oils was strongly dependent on the method used, suggesting that different groups of compounds contribute to distinct antioxidant mechanisms. EOs presented a high ability to inhibit lipid peroxidation (Figure 1A), with inhibition values between 20 and 40% in the tested concentrations of 0.25 and 0.50 mg/mL. The high antioxidant activity observed in the lipid peroxidation inhibition method could be related to the chemical composition of EOs, namely to the presence of a high content of oxygenated monoterpenes [55] and their diversity in their constituents in amount as well as in different compounds present in spring and autumn EOs. Indeed, sample Y1A generally exhibited the strongest inhibitory effect and a lower IC_50_ value (IC_50_ = 0.854 ± 0.04 mg/mL), compared with Y1S (IC_50_ = 1.57 ± 0.08 mg/mL), confirming its higher effectiveness in this assay. This behaviour may be associated with a distinct chemical profile rich in oxygenated monoterpenes, specifically ketones such as isopulegone (35.9%), isomenthone (24.9%), pulegone (13.8%), and menthone (4.6%), compounds previously reported to contribute to membrane protection and inhibition of lipid peroxidation.

Regarding radical scavenging activity (Figure 1B and Table 3), Y1S exhibited a high antioxidant activity with a significantly lower IC_50_ value (IC_50_ = 27.99 ± 1.40 mg/mL) than Y1A (36.76 ± 1.84 mg/mL), indicating a greater ability to neutralize free radicals (*p* < 0.05). The higher scavenging activity of Y1S may be related to its higher contents of isopulegone (44.7%) and pulegone (13.8%), together with the presence of minor constituents such as thymol, carvacrol, and caryophyllene oxide, that have been reported to contribute to the synergistic effect on radical scavenging activity. Nevertheless, the antioxidant response is unlikely to be determined by a single compound, but rather by synergistic interactions among the different constituents of the essential oil. This is consistent with recent findings proposed by Juncos, et al. [56], who established that specific pulegone-to-ketone ratios serve as definitive predictive benchmarks for evaluating baseline radical scavenging efficiency in industrial Lamiaceae matrices. These findings highlight the importance of the overall phytochemical profile in determining the free-radical scavenging capacity of *C. nepeta* essential oils.

In the ferric reducing power assay (Figure 1C and Table 3), Y2A exhibited the highest reducing capacity, with a significantly lower IC_50_ value (18.31 ± 1.20 mg/mL) than Y1S (25.17 ± 1.26 mg/mL), Y1A (32.16 ± 1.61 mg/mL) and Y2S (40.88 ± 3.81 mg/mL) (*p* < 0.05), indicating a superior electron-donating ability. This result is consistent with the concentration-dependent response observed in Figure 1C, where Y2A reached approximately 75% reducing activity at 30 mg/mL. The enhanced reducing power of Y2A coincided with a distinctive phytochemical profile characterised by significantly higher levels of 1,8-cineole (16.30%), isomenthone (31.98%), menthol (17.82%), α-terpineol (0.73%), anethole (4.36%), and β-caryophyllene (0.61%). In contrast, Y2S, despite containing the highest proportions of isopulegone (49.69%) and elevated pulegone levels (13.47%), exhibited the weakest reducing activity. Results suggest that ferric reducing capacity is more closely associated with the abundance of oxygenated monoterpenes and their synergistic interactions than with the concentration of individual ketones. Overall, the results highlight the influence of seasonal variation on the phytochemical composition and electron-transfer properties of *C. nepeta* essential oils.

The statistical analysis confirmed the observed variations in biological activity between the different harvest years and seasonal conditions. As indicated by the distinct lowercase letters (Table 3 and throughout Figure 1), one-way ANOVA followed by Tukey’s post hoc test (*p* ≤ 0.05) demonstrated that the differences in antioxidant activity among the samples were statistically significant. In the DPPH radical scavenging assay, the first-year spring sample (Y1S) showed significantly greater activity, presenting a lower IC_50_ value (27.99 ± 1.40 mg/mL) than the autumn sample Y1A (36.760 ± 1.838 mg/mL). In contrast, the iron reducing power and lipid peroxidation assays indicated a significant effect of environmental conditions, particularly during the exceptionally warm autumn of the second year. The Y2A sample exhibited a significantly higher reducing capacity (Figure 1C), clearly differing from both Y1S and Y1A.

Overall, these statistical findings indicate that the climate-related shift from a ketone-dominant chemical profile to one richer in alcohols and oxides influenced the antioxidant behaviour of the essential oils. This variation appears to affect the balance between radical-scavenging activity and electron-donating capacity, particularly under extreme climatic conditions that may influence the conventional IC_50_ response patterns. Briefly, the EOs main antioxidant mechanism was the inhibition of lipid peroxidation, suggesting their high ability to protect lipid substrates. The observed differences between the EOs samples are clearly modulated by the interaction between the plant’s phenology and the climatic extremes of the Alentejo region, where specific events, such as intense heatwave conditions, directly impacted the EOs’ redox potential.

A previous study conducted by our research group involving the EO of *C. nepeta* species harvested during the same season as sample Y1A highlighted the antioxidant activity of this species with IC_50_ values of 0.854, 26.84, and 32.60 mg/mL in lipid peroxidation inhibition, reducing power, and DPPH radical scavenger methods, respectively [7]. Antioxidant activities of *C. nepeta* EO from the south of Portugal have shown that these EOs possess antioxidant capacity, both to scavenge free radicals and to reduce Fe^3+^ and inhibit lipid oxidation [57].

The antioxidant mechanisms of *C. nepeta* EOs are distinct across the three assays, governed by the structural features of their dominant *p*-menthane derivatives. In the DPPH assay, activity relies on the ability of monoterpene ketones (pulegone and isopulegone) to act as hydrogen donors; although non-phenolic, their allylic hydrogens can be abstracted to stabilize the DPPH• radical. In the LPO assay, the EOs demonstrate a high protective effect on lipid substrates (inhibition up to 40% at 0.5 mg/mL), likely due to the lipophilic nature of the monoterpene hydrocarbon skeletons, which allows for effective integration into the β-carotene/linoleic acid emulsion. Finally, the Iron Reducing Power results, particularly for Y2A, are explained by the structure-activity relationship (SAR) of menthol; its hydroxyl group facilitates the donation of electrons to transform Fe^3+^ into Fe^2+^, a mechanism that is enhanced in this sample due to the heat-induced accumulation of this alcohol [7,10,42].

The presence of some oxygenated monoterpenes, such as 1,8-cineole (up to 16.30% in Y2A) or hydrocarbon monoterpenes, such as α-terpinene, ϒ-terpinene, 4-terpineol, and limonene (up to 4.15% in Y2S) in the EOs chemical composition is described as responsible for promoting the increase in antioxidant activity [57]. Even at lower concentrations, these hydrocarbons can significantly modulate the redox potential of the major ketones, providing a more complex and robust protective profile against oxidative stress. Pulegone is also described to exhibit several bioactivities that can promote the antioxidant activity of these essential oils [10]. EO of *C. nepeta* from Algeria was tested by DPPH free radical scavenging and reducing power assays and showed low activity, lower than synthetic standards BHT and BHA [19]. EO from Morocco presented similar results, and the authors explained the lower activity as EO is poor in phenolic compounds and due to the high content of 1,8-cineole (>40%) in its chemical composition [10].

Our findings suggest that the chemical plasticity of *C. nepeta* in response to Alentejo’s climatic extremes, ranging from the wettest spring to the warmest autumn on record, not only alters its terpene profile but also modulates its protective antioxidant capacity, reinforcing its potential as a robust source of natural antioxidants as food antioxidants as well as natural protectors against oxidative stress disorders.

### 2.3. Antimicrobial Properties of C. nepeta EOs

The results of the disc-diffusion assay are reported in Table 4. EOs presented a large spectrum of antibacterial activity against *Gram*-positive and *Gram*-negative pathogenic bacteria, although the magnitude of inhibition differed considerably among specific microorganisms and harvest periods.

A highly significant finding was the occurrence of total inhibition (t. i.) events, which highlighted the targeted efficacy of specific seasonal profiles. The antimicrobial capacity of these EOs is remarkable and can be attributed to the presence of several chemical groups, such as oxygenated monoterpenes and hydrocarbons [8,15,28,36]. For example, the oil with the highest proportion of isopulegone (49.69%) in Y2S, induced by the exceptional precipitation levels of that season, appears to provide targeted efficacy against certain Proteobacteria, notably causing total inhibition of *M. morganii* LFG 1008. In contrast, the autumn profile of the second year (Y2A), which was rich in isomenthone (31.98%), menthol (17.82%), and 1,8-cineole (16.30%), sample Y2A, due to thermal extremes, appears more effective against *Gram*-positive strains *E. faecalis*, *S. aureus*, *and S. epidermidis*. Complete growth inhibition was observed with the Y2A essential oil against *Enterococcus faecalis* ATCC 29212, *E. faecalis* LFG 1001, *Staphylococcus aureus* LFG 1007, and *Staphylococcus epidermidis* ATCC 12228. In addition, the spring-harvested oil from the first year (Y1S) induced total inhibition of *S. aureus* ATCC 29213.

Among the Gram-positive bacteria for which inhibition zones could be determined, considerable differences in susceptibility were recorded between strains and seasonal chemotypes. The Y2A oil demonstrated particularly pronounced activity against *S. aureus* ATCC 29213, producing an inhibition zone of 26.2 ± 1.16 mm, closely comparable to that obtained with tetracycline hydrochloride (29.2 ± 0.4 mm). Similarly, the Y1A sample exhibited strong antibacterial activity against the clinical isolate *S. aureus* LFG 1007, with an inhibition zone of 20.8 ± 2.1 mm. Regarding *Gram*-negative bacteria, the essential oils generally displayed moderate to marked antibacterial effects. *Escherichia coli* ATCC 25922 was among the most susceptible reference strains, with inhibition zones ranging from 16.7 ± 1.8 mm to 24.0 ± 5.7 mm for the Y2A EO. Clinical isolates of *M. morganii* and *Salmonella enteritidis* also showed considerable susceptibility, particularly towards Y1S and Y2A EOs. Notably, *Proteus mirabilis* LFG 1004 exhibited inhibition zones ranging from 11.0 ± 1.7 mm to 12.3 ± 0.8 mm across all seasonal samples, values that were significantly greater than those produced by tetracycline hydrochloride (7.3 ± 0.7 mm). In contrast, the reference strain *Pseudomonas aeruginosa* ATCC 27853 demonstrated marked resistance to most oils, with no visible inhibition (w. i.) detected for Y1S, Y2S, or Y2A, the clinical isolate P. aeruginosa LFG1002 displayed a notable and unique susceptibility to the thermal-stress-modulated Y2A oil, yielding an inhibition zone of 19.7 ± 4.7 mm, which slightly exceeded the activity of the standard tetracycline hydrochloride (17.6 ± 0.5 mm). The enhanced antibacterial activity observed for the Y2A sample may be associated with its altered phytochemical profile, characterized by an increased abundance of oxygenated monoterpenes, including menthol, isomenthone, and 1,8-cineole, likely resulting from climatic and thermal stress conditions.

Overall, the disc diffusion assays demonstrate that the antibacterial activity of *C. nepeta* essential oils is strongly influenced by seasonal and environmentally driven variations in chemical composition, together with the intrinsic susceptibility of the bacterial strains tested. These findings further support the potential application of these essential oils as natural antimicrobial agents.

The microdilution antimicrobial assay (Table 5) provided important quantitative insights that corroborated the disc-diffusion trends. Overall, the typical spring sample (Y1S) displayed the most consistent and potent baseline antibacterial activity, yielding the lowest MIC values across the majority of the tested microorganisms, with values ranging from 465 to 1767 µg/mL, when compared with Y2S, Y1A, and Y2A EOs with values ranging from 1023 - >2046 μg/mL, 792 - >3956 μg/mL, and 1001–4550 μg/mL, respectively. The variability observed in MIC values, where Y1S was generally more effective than the autumn essential oils (Y1A and Y2A), suggests that, although extreme weather events favor the prevalence of certain components, the balanced “ketone-type” profile of a typical spring (Y1S) may offer a more robust multi-target antibiotic effect.

The subsequent evaluation of the Minimum Bactericidal Concentration (MBC) through subculturing from the non-turbid wells onto solid media revealed a distinct mode of action. For all evaluated seasonal EOs and tested strains, no total clearance of colonies was observed at the tested concentrations, or the MBC values heavily exceeded the MIC thresholds. This rigorous experimental confirmation establishes that the essential oils of *C. nepeta* act primarily through a broad-spectrum bacteriostatic mechanism rather than a bactericidal one, effectively arresting bacterial proliferation and metabolic growth without causing immediate cell lysis.

The obtained results demonstrate that the essential oils (EOs) of *C. nepeta* exhibited a broad bacteriostatic spectrum against both *Gram*-positive and *Gram*-negative bacteria, although with marked variations according to the harvesting season and year. Overall, the Y1S sample displayed the strongest antibacterial activity, presenting the lowest MIC values for the majority of the tested microorganisms, particularly against *Gram*-positive strains such as *S. aureus* ATCC 29213 and *S. epidermidis* ATCC 12228. These findings suggest that seasonal and environmental factors significantly influence the chemical composition of the oils and, consequently, their antimicrobial effectiveness. As expected, tetracycline hydrochloride, used as a standard, showed bacteriostatic activity with values ranging from 1.0 µg/mL to 500 µg/mL. Despite the seasonal differences, *C. nepeta* EOs showed a relevant antibacterial effect, mainly against *Gram*-negative clinical isolates, including *Morganella morganii*, *Pseudomonas aeruginosa*, and *Escherichia coli*. Moreover, the Y2A EO, characterized by high levels of isomenthone (31.98%), menthol (17.82%), and 1,8-cineole (16.30%), compounds likely promoted by thermal stress conditions, demonstrated enhanced activity against *P. aeruginosa* LFG1002, with MIC values ranging between approximately 837 and 1365 µg/mL, in Y1S and Y2A, respectively. This result suggests that thermally induced modifications in the secondary metabolism of the plant may enhance the production of bioactive monoterpenes associated with antibacterial activity.

Conversely, specific *Gram*-positive clinical isolates (LFG) demonstrated reduced susceptibility when compared directly to their respective reference strains (ATCC) under identical seasonal oil treatments. For instance, the clinical isolate *Enterococcus faecalis* LFG 1001 exhibited a higher tolerance to the Y1A oil (MIC > 3956 µg/mL) than its reference counterpart *E. faecalis* ATCC 29212 (MIC > 1978 µg/mL). This variation suggests that the antimicrobial efficacy of the oils is influenced not only by bacterial cell wall structure but also by specific interactions between the volatile bioactive constituents and cellular targets of different bacterial species, as well as by specific strain-level resistance mechanisms and target variations found in wild-type clinical isolates [11,36]. Typically, *Gram*-negative bacteria exhibit greater resistance to essential oils due to their outer wall, composed of lipopolysaccharides, surrounding the peptidoglycan cell wall, which constitutes a barrier to essential oils [58]. Nevertheless, results of this study demonstrate that *C. nepeta* EOs, particularly those modulated by extreme seasonal events, can effectively bypass or disrupt this barrier in specific clinical isolates. It is important to note that the strains used in the study are clinical isolates, pathogenic and/or opportunistic pathogens, which mostly exhibit resistance to synthetic antimicrobials. Therefore, it is necessary to research new compounds to which these microorganisms are sensitive and which can be subsequently applied in therapy. These results suggest that changes in the chemical profiles of EOs, due to metabolic alterations driven by extreme weather events, do not compromise their antimicrobial efficacy and may even redirect it to specific clinical targets.

Despite this predominant bacteriostatic nature of *C. nepeta* EOs, differences were observed in the antimicrobial ability of EOs depending on the season and harvest year.

Spring EOs revealed higher antimicrobial activity than both autumn EOs. This fact may be related to the higher pulegone content (up to 13.83% in Y1S) observed in spring EO [2]. Flamini, et al. [15] reported that the antimicrobial potential of *C. nepeta* EO is due almost exclusively to pulegone, one of the major components [15]. However, the antimicrobial effectiveness of EOs is not only due to the major component of EOs but also to the synergistic interaction between minor and major EO components, such as the presence of limonene (up to 4.15%) and *trans*-isopulegone (up to 9.20%) [10].

High antimicrobial activity and wide spectra of *C. nepeta* observed in this study, even against drug-resistant strains, can be correlated with the specific dynamics of their high content of oxygenated monoterpenes and sesquiterpenes [23,59] and their major components on the bacterial architecture. Major components presented in studied EOs, such as 1,8-cineole, isomenthone, isopulegol, and menthol and their respective isomers, are reported with high antibacterial properties, with the ability to disrupt bacterial membrane integrity, alter permeability, and interfere with essential cellular processes, ultimately leading to growth inhibition [11,36,60].

The EOs’ mechanism of action seems not to be attributed to one specific action but to several targets in the cell. It is generally known that antimicrobial potential may be correlated to the reported ability of EOs to disrupt the permeability barrier of cell membrane structures. The lipophilic character of their hydrocarbon skeleton and the hydrophilic character of their functional groups are critical for the antimicrobial action of EOs [16,21,23,24,44].

In the last few decades, there has been an increase in the occurrence of infectious diseases worldwide, and pathogenic bacteria have usually developed resistance to the recently used antimicrobial drugs. Thus, the application of essential oils due to the synergistic effect of their components could help decrease multiple antimicrobial resistance and be suitable as complementary medicine [8,10,36,58]. In a global context of rising antimicrobial resistance, the chemical plasticity of *C. nepeta* EOs in response to the Alentejo’s environmental extremes (ranging from severe heatwaves to record rainfall) provides a diverse library of volatile compounds. Our findings suggest that these climate-induced metabolic shifts do not diminish bioactivity; rather, they can generate a diverse library of volatile compounds and specialized profiles (such as the Y2A chemotype) that effectively target multidrug-resistant (MDR) strains. The microorganisms selected for this study are of significant concern in both clinical and food safety contexts. *Staphylococcus aureus* and *Enterococcus faecalis* are major causes of nosocomial infections, while *Morganella morganii* and *Pseudomonas aeruginosa* are opportunistic pathogens frequently associated with multidrug resistance (MDR) in hospital settings. From a food industry perspective, the ability of these EOs to inhibit *Escherichia coli* and *Salmonella* spp. highlights their potential as natural preservatives to prevent foodborne outbreaks and extend shelf life, offering a bio-based alternative to synthetic additives [30,36,54,61].

## 3. Materials and Methods

### 3.1. Chemicals

Analytical standards for chromatography (>99%) were purchased from Sigma-Aldrich (St. Louis, MO, USA) and Extrasynthese (Genay, France). Ascorbic acid (>99%), 2,2-diphenyl-1-picryl-hydrazyl (DPPH) (95%), β-carotene (95%), linoleic acid (99%), nystatin, and tetracycline hydrochloride and Whatman sterile filter paper discs (Ø = 6 mm) were purchased from Sigma-Aldrich (St. Louis, MO, USA). Culture media were obtained from Merck KGaA (Darmstadt, Germany). Oxoid microbiology media and antimicrobial susceptibility discs were purchased from Thermo Fisher Scientific (Waltham, MA, USA). All other chemicals and solvents of analytical or HPLC grade were obtained from Sigma-Aldrich (St. Louis, MO, USA).

### 3.2. Isolation of Essential Oils

Wild-grown plants of *Calamintha nepeta* subsp. *nepeta* were collected in spring and in autumn in Évora, Alentejo region (38°31′39.4″ N, 8°0′59″ W) in two different years. Plant material of spring (May) and autumn (October) were identified by Professor Marízia Menezes (University of Évora) and deposited in the herbarium of Aromatic Plants of the University of Évora, with the accession numbers between HPAMT_UE 000005–HPAMT_UE 000008. Aerial parts were separated and shade-dried at room temperature until constant weight (approx. 72 h).

Samples were collected during consecutive years to capture the chemical variability associated with different phenological stages and climatic conditions. Meteorological data for each period were retrieved from the official reports of the Portuguese Institute for Sea and Atmosphere (IPMA). The region presents a Mediterranean climate classified as Csa (hot-summer Mediterranean climate) according to the Köppen–Geiger classification [62]. The sampling periods (summarized in Table 6) were characterized as follows:

The Y1S (Year 1 Spring) was characterized as the coldest spring since 1993 [40]. While the quarter was rainy (154% of normal), the period recorded the 2nd wettest March in mainland Portugal in the last 50 years, and the lowest May average temperature in the last 20 years, and with the lowest minimum temperature in the last 30 years, including late-season snow and frost events in Portugal at altitudes above 900 m. Leading up to the harvest, temperatures and precipitation levels were below the historical average (13.17 °C and 29.6 mm), establishing a low-temperature baseline for the spring profile. Samples were collected during the vegetative stage.

The Y1A (Year 1 Autumn was characterized by an average temperature 0.76 °C above normal, making it the 7th warmest autumn in the last 30 years. This period included a documented funnel cloud (tornado F0/T1) in Montemor-o-Novo, providing a source of intense mechanical stress (thigmomorphogenesis), contributing to an unstable climatic profile [40]. *C. nepeta* samples were collected during the flowering stage.

The Y2S (Year 2 Spring) was characterized by a spring atypically cold and extremely rainy, although it began with a meteorological drought in the Lower Alentejo region, and transitioned into the rainiest spring in the last 15 years, with average rainfall levels for the quarter corresponding to 164% of the normal value [40], representing a significant abiotic hydric stressor. Samples were collected during the vegetative stage.

The Y2A (Year 2 Autumn) was a season with the 2nd warmest and 6th rainiest autumn since 1931 (being the 3rd wettest since 1972). It was specifically marked by a severe heatwave in October (the most significant since 1941) and record-high minimum temperatures [40], creating a unique thermal stress environment. Samples were collected during the flowering stage.

For each sampling period, six replicates of approximately 150–200 g (dry weight) of aerial parts were subjected to hydrodistillation [8] for 3 h in a *Clevenger*-type apparatus, according to the European Pharmacopoeia. EO yield (%) was calculated on a dry-weight basis.

Relative density ($d_{20}^{20}$) was determined by the gravimetric method using micro-pycnometry adaptation; briefly, the mass of 100 µL of EO was measured at 20 °C and compared to the mass of an equal volume of distilled water. Measurements were performed in six replicates using a precision analytical balance (±0.1 mg).

The refractive index (ɳd^20^) was measured by a refractometer (Leica Abbe Mark II, Model 10481; Leica Microsystems, Wetzlar, Germany). Essential oils were stored at 4 °C before chemical analysis and biological study.

### 3.3. Chemical Characterization of Essential Oils

EOs analysis was performed by gas chromatography with a flame ionization detector (GC-FID) and gas chromatography-mass spectrometry (GC-MS), following adapted procedures from Arantes, et al. [8].

GC-FID analysis was performed on a Shimadzu Nexis GC-2030 equipped with an AOC20i autoinjector plus LabSolutions 5.92 software (Shimadzu Corporation, Kyoto, Japan). Analysis were conducted on a non-polar fused silica column (30m × 0.25 mm id, film thickness 0.50 μm) Zebron ZB-5HT Inferno™ (Phenomenex, Torrance, CA, USA), under the following conditions: oven temperature programme 40–110 °C (2 °C/min), 110–220 °C (3 °C/min), and 220–280 °C (10 °C/min), injector temperature 250 °C, detector temperature 310 °C, carrier gas flow rate 1.6 mL/min He and split ratio 25:1. 1 µL of diluted EO was injected, and the analyses were carried out in triplicate. GC-MS analysis was performed on a Shimadzu GCMS-QP2010 Series system (Shimadzu)gas chromatograph, equipped with a detector model Polaris Q (E. I. quadrupole) and using the same Zebron ZB-5HT Inferno™ column.

Compounds were identified based on their retention indices and their mass spectra. Retention indices were determined systematically by linear interpolation relative to the retention time of a homologous series of C8–C40 *n*-alkanes, using the Van den Dool and Kratz equation, and they were compared with those of authentic standards and literature data [2,8,41,42]. Acquired mass spectra were compared with reference spectra from the database NIST 11 (National Institute of Standards and Technology) library. Quantitative data of individual components of EO were determined using relative percentage abundance and normalization method without the use of response factors for flame ionization detection. Percentage values are the mean of the peak areas of three injections per sample.

### 3.4. Antioxidant Properties of Essential Oils

#### 3.4.1. Lipid Peroxidation Inhibition Assay (LPO)

The capacity of the essential oils (EOs) to inhibit lipid peroxidation was evaluated using the β-carotene–linoleic acid bleaching assay [7,8]. Briefly, a β-carotene/linoleic acid emulsion was prepared and aliquoted into the wells of a microplate. EO solutions (0.04–2.50 mg/mL, prepared in ethanol containing 5% Tween 20) and ascorbic acid (0.06–4.00 mg/mL, prepared in distilled water) were added to the reaction mixture. Absorbance was measured at 490 nm immediately after sample addition (t = 0 h) and after incubation at 50 °C for 2 h [7,55]. A control containing the solvent system without an antioxidant was included. All assays were performed in triplicate.

Lipid peroxidation (LPO) inhibition was calculated using the following equation:

LPO (%) = [(ΔAc − ΔAs)/ΔAc] × 100,

where ΔAc is the difference between absorbance at 0 h and 2 h for the control, and ΔAs represents the difference between absorbance at 0 h and 2 h for samples or standards.

#### 3.4.2. DPPH Radical Scavenging Assay

Free radical scavenging activity was determined based on the bleaching of a purple-coloured ethanol solution of the stable radical 2,2-diphenyl-1-picryl-hydrazyl (DPPH•). This assay evaluates the ability of the EOs to act as hydrogen atoms or electron donors in the transformation of DPPH• into its non-radical form DPPH-H.

Antioxidant activity of the EOs (0.5–52.0 mg/mL in ethanol with 5% Tween 20), and ascorbic acid (0.25–65.0 μg/mL in water) was determined according to a method described by Tepe, et al. [55], with minor modifications [7].

The DPPH radical scavenging activity (inhibition %) was calculated using the equation:

Inhibition (%) = [(AC − AS)/AC] × 100,

where AC is the absorbance of the control, and AS is the absorbance of the sample.

#### 3.4.3. Iron Reducing Power Assay

The ferric reducing power of the essential oils (EOs) was evaluated using a microplate-adapted method described by Arantes et al. [8]. Briefly, 200 μL of EO solutions (0.4–60.0 mg/mL in ethanol containing 5% Tween 20) were mixed with 50 μL of Tween 20 in microtubes. For the reference standards, the same volume of solvent was used to maintain matrix consistency. Subsequently, 200 µL of phosphate buffer (0.2 M, pH 6.6) and 200 µL of potassium ferricyanide (1%) were added. After homogenisation, the mixtures were incubated at 50 °C for 20 min. The reaction was stopped by adding 200 μL of 10% trichloroacetic acid, followed by centrifugation at 3000 rpm for 10 min. A reagent blank containing all assay components except EO or standard was used for baseline correction. Ascorbic acid (0.4–60.0 mg/mL in water) was used as the reference antioxidant. Aliquots (50 μL) of the resulting supernatants were transferred to a 96-well microplate and mixed with 50 μL of distilled water and 100 μL of 0.1% ferric chloride (FeCl_3_). Absorbance was measured at 700 nm using a Multiskan™ Go UV–Vis microplate reader (Thermo Fisher Scientific, Waltham, MA, USA). Increased absorbance values were taken as indicative of greater reducing power. All measurements were performed in triplicate.

### 3.5. Antimicrobial Properties of Essential Oils

#### 3.5.1. Test Microorganisms

In vitro, the antimicrobial activity of essential oil was tested following the CLSI Guidelines using two methods: the paper disc diffusion assay [63] and the Minimal Inhibitory Concentrations (MICs) for susceptible strains [64]. The antibacterial activity was tested on 13 different bacteria, provided by American Type Culture Collection (ATCC) and clinical isolates obtained from a medical diagnostic Laboratory in the Évora region (LFG). Bacterial strains were activated in Nutrient Agar at 37 °C for 24 h before testing. The susceptibility of the strains was interpreted according to the clinical breakpoints defined in the CLSI M100-ED36:2026 [65].

#### 3.5.2. Antimicrobial Screening

Disc diffusion assays were performed in Mueller-Hinton agar plates, for bacteria, using sterile filter paper discs (∅ = 6 mm) impregnated with 5 µL of EO, as described in a previous study [8]. Specific antimicrobial discs were used: tetracycline chloride (30 μg/disc) for bacteria-positive control. A sterilized physiological saline solution (5 μL) was used as a negative control sample. To minimize EO volatilization and allow for pre-diffusion, plates were kept at 4 °C for 2 h before being incubated at 37 °C/24 h. The diameter of the inhibition zones (mm) was measured using a Fisher-Lilly Antibiotic Zone Reader (Fisher Scientific).

#### 3.5.3. Evaluation of Minimum Inhibitory Concentration (MIC) and Minimum Bactericidal Concentration (MBC)

The Minimum Inhibitory Concentration (MIC) was determined using the broth microdilution method in sterile 96-well microplates, following CLSI M07-A10 standards [64], with adaptations for essential oils. The EOs were initially solubilized in sterile culture media supplemented with DMSO (6%, *v*/*v*) and Tween 20 (2.5%, *v*/*v*) [8]. Briefly, 100 µL of each EO (serial dilutions ranging from 0.03 to 5.00 µL EO/mL, subsequently converted to mg/mL) was mixed with 100 µL of the microbial inoculum (10^6^ UFC/mL in sterile medium), resulting in a final concentration of 3% DMSO and 1.25% Tween 20. Solvent control wells were included to confirm that these concentrations did not affect microbial growth. To ensure these solvents did not interfere with microbial growth, two control groups were maintained: a positive growth control (6% DMSO and 2.5% Tween 20 supplemented medium + inoculum) and a negative control without inoculum, at well final concentrations of 3% and 1.25% (*v*/*v*) of DMSO and Tween 20, respectively. These controls confirmed that the final concentrations of DMSO and Tween 20 had no inhibitory or stimulatory effects on the tested strains. Tetracycline hydrochloride (0.98–1000 µg/mL in sterile water) was used as the reference antibiotic. Plates were incubated at 37 °C for 18 h, and MIC values, defined as the minimum concentration of the test samples that inhibits the selected organism’s growth, were determined by visual inspection of the absence of turbidity. All experiments were performed in triplicate.

To determine the Minimum Bactericidal Concentration (MBC), 5 µL from the wells showing no visible growth were subcultured onto solid media and incubated at 37 °C for 24 h. The MBC value was defined as having the lowest concentration with no visible colonial growth.

### 3.6. Statistical Analysis

All data were expressed as the mean ± standard deviation of different measurements. Statistical analysis of the data was performed using one-way ANOVA to determine statistically significant differences between the seasonal harvests and climatic events. Differences between samples obtained at the *p* ≤ 0.05 level were considered significant, and multiple comparisons of means were analysed using the Tukey test. Statistically significant differences between treatments are indicated in the tables and figures by different lowercase letters. Analyses were performed using IBM SPSS ^®^ Statistics for Windows, Version 24.0 (IBM Corp., Armonk, NY, USA).

## 4. Conclusions

The purpose of this study was to evaluate the metabolic resilience of *C. nepeta* under extreme abiotic stress. The results demonstrated that the chemical profile and biological potential of essential oils (EOs) from *Calamintha nepeta* subsp. *nepeta* are highly plastic and strongly influenced by local edaphoclimatic conditions, acting as a functional response to record-breaking climatic events rather than mere seasonal fluctuations. The transition from a typical “ketone-type” profile during climatically stable periods to profiles dominated by biosynthetic precursors or saturated derivatives under extreme precipitation (Y2S) or thermal and mechanical stress (Y2A) was associated with significant changes in EO bioactivity.

The results indicate that *C. nepeta* exhibits marked metabolic plasticity in response to environmental conditions. Typical spring conditions favoured the accumulation of p-menthane ketones (isopulegone and pulegone), which were associated with higher antimicrobial activity against multidrug-resistant (MDR) clinical isolates. In contrast, extreme climatic conditions were associated with shifts in essential oil composition, leading to chemotypes enriched in isopulegone/pulegone or in isomenthone and menthol.

In conclusion, the present study demonstrates that extreme climatic conditions strongly influence the chemical composition of *C. nepeta* essential oils, resulting in significant variations in their biological activities. These results highlight the importance of detailed chemical characterisation of essential oils for the accurate validation and interpretation of their biological and pharmacological properties, thereby ensuring their reproducibility, efficacy, and standardisation for therapeutic and industrial applications.

These findings reinforce the resilience of *C. nepeta* as a source of bioactive compounds and underscore the critical need to consider climate-induced metabolic shifts in the standardization of essential oils. Monitoring local weather conditions is, therefore, as crucial as the harvest season for the industrial application of these oils in the pharmaceutical and food industries.

## Figures and Tables

**Table 1:** Extraction yield %, density, and refractive index (ηd) of *Calamintha nepeta* EOs.

EO	Extraction Yield % (*v*/*w*)	Density	EO (ηd^t^)
Y1S	0.50 ± 0.05 ^a^	0.927 ± 0.001 ^a^	1.47 ± 0.01 ^a^
Y1A	0.61 ± 0.08 ^a^	0.989 ± 0.001 ^a^	1.47 ± 0.01 ^a^
Y2S	0.44 ± 0.03 ^b^	0.930 ± 0.010 ^a^	1.46 ± 0.01 ^a^
Y2A	0.80 ± 0.04 ^c^	0.937 ± 0.003 ^a^	1.47 ± 0.01 ^a^

Y1S—Year 1 Spring; Y1A—Year 1 Autumn; Y2S—Year 2 Spring; Y2A—Year 2 Autumn. Values in columns with different letters show statistically significant differences based on ANOVA and the Tukey test at *p* ≤ 0.05.

**Table 2:** Chemical composition of *C. nepeta* EOs.

Compound	RI_exp._ **	RI_ref_ ***	Area (%) *			
			Y1S	Y1A	Y2S	Y2A
α-Pinene	930	932 [2]	0.11 ± 0.02 ^a^	0.22 ± 0.01 ^b^	0.33 ± 0.01 ^c^	0.21 ± 0.01 ^b^
Camphene	942	945 [2]	-	0.05 ± 0.01 ^a^	0.02 ± 0.00 ^b^	0.06 ± 0.01 ^a^
Sabinene	970	966 [2]	0.09 ± 0.03 ^a^	0.35 ± 0.01 ^b^	0.37 ± 0.00 ^b^	0.17 ± 0.03 ^c^
β-Pinene	971	972 [2]	0.15 ± 0.05 ^a^	0.40 ± 0.01 ^b^	0.71 ± 0.01 ^c^	0.37 ± 0.05 ^b^
β-Myrcene	990	983 [2]	-	0.30 ± 0.01 ^a^	0.34 ± 0.01 ^a^	0.31 ± 0.01 ^a^
α-Phellandrene	1002	1004 [41]	0.08 ± 0.01 ^a^	0.06 ± 0.01 ^b^	0.11 ± 0.00 ^c^	0.08 ± 0.00 ^a^
δ-3-Carene	1007	1011 [41]	-	-	-	0.14 ± 0.02
*p*-Cymene	1021	1014 [2]	0.18 ± 0.02 ^a^	0.17 ± 0.01 ^a^	0.14 ± 0.01 ^a^	0.51 ± 0.04 ^b^
Limonene	1026	1023 [2]	0.34 ± 0.14 ^a^	0.37 ± 0.03 ^a^	0.82 ± 0.13 ^b^	0.13 ± 0.04 ^a^
1,8-Cineole	1027	1023 [2]	**6.88 ± 0.65** ^a^	**8.78 ± 0.26** ^b^	**9.84 ± 0.12** ^c^	**16.30 ± 0.23** ^d^
*E*-β-Ocimene	1038	1037 [2]	-	-	0.05 ± 0.00 ^a^	0.09 ± 0.01 ^a^
ϒ-Terpinene	1044	1049 [2]	0.16 ± 0.05 ^a^	0.06 ± 0.01 ^b^	0.03 ± 0.00 ^b^	0.03 ± 0.00 ^b^
*trans*-Sabinene hydrate	1064	1053 [2]	0.07 ± 0.01 ^a^	0.18 ± 0.01 ^b^	0.09 ± 0.00 ^c^	0.16 ± 0.00 ^d^
*cis*-Sabinene hydrate	1083	1084 [2]	0.15 ± 0.02 ^a^	0.27 ± 0.10 ^a^	0.24 ± 0.01 ^a^	1.51 ± 0.13 ^b^
α-Terpinolene	1086	1076 [2]	0.07 ± 0.01 ^a^	-	-	0.03 ± 0.01 ^a^
Linalool	1100	1084 [2]	0.07 ± 0.01 ^a^	0.13 ± 0.02 ^b^	0.12 ± 0.00 ^b^	0.43 ± 0.02 ^c^
Camphor	1139	1143 [42]	0.09 ± 0.01 ^a^	0.19 ± 0.04 ^b^	0.15 ± 0.00 ^b^	0.18 ± 0.00 ^b^
Isopulegol	1142	1144 [8]	-	0.06 ± 0.00 ^a^	0.04 ± 0.00 ^a^	0.09 ± 0.01 ^a^
Menthone	1150	1132 [2]	**4.63 ± 0.19** ^a^	3.61 ± 0.14 ^b^	**5.50 ± 0.01** ^c^	2.35 ± 0.04 ^d^
*neo*-Menthol	1155	1151 [2]	-	0.35 ± 0.03 ^a^	0.31 ± 0.01 ^a^	0.04 ± 0.00 ^b^
Isomenthone	1160	1159 [42]	**17.41 ± 0.11** ^a^	**24.87 ± 0.01** ^b^	**7.45 ± 0.03** ^c^	**31.98 ± 0.24** ^d^
*neo*-*iso*-Isopulegol	1164	1161 [2]	1.53 ± 0.18 ^a^	1.80 ± 0.01 ^a^	4.36 ± 0.02 ^b^	-
Terpinen-4-ol	1168	1177 [41]	0.37 ± 0.05 ^a^	0.74 ± 0.08 ^b^	0.49 ± 0.05 ^a^	0.84 ± 0.08 ^b^
Isopulegone	1178	1177 [41]	**44.68 ± 0.76** ^a^	**35.90 ± 2.28** ^b^	**49.69 ± 0.04** ^c^	**10.03 ± 0.18** ^d^
Menthol	1186	1178 [41]	3.06 ± 0.11 ^a^	**5.32 ± 0.42** ^b^	3.35 ± 0.07 ^a^	**17.82 ± 0.54** ^c^
α-Terpineol	1188	1190 [41]	0.32 ± 0.01 ^a^	0.35 ± 0.03 ^a^	0.51 ± 0.01 ^b^	0.73 ± 0.06 ^c^
Piperitone	1216	1254 [41]	0.06 ± 0.00 ^a^	-	0.03 ± 0.01 ^a^	-
*trans*-Piperitone oxide	1233	1228 [2]	0.11 ± 0.03 ^a^	0.11 ± 0.01 ^a^	-	0.10 ± 0.00 ^a^
Pulegone	1235	1234 [41]	**13.83 ± 0.17** ^a^	**8.44 ± 0.02** ^b^	**13.47 ± 0.03 ^c^**	**5.10 ± 0.05** ^d^
Carvone	1242	1242 [41]	0.08 ± 0.01 ^a^	0.06 ± 0.01 ^a^	0.30 ± 0.01 ^b^	0.85 ± 0.01 ^c^
Isopulegyl acetate	1257	1254 [2]	0.30 ± 0.02 ^a^	0.11 ± 0.02 ^b^	0.02 ± 0.00 ^c^	0.04 ± 0.00 ^c^
Menthyl acetate	1280	1296 [41]	0.07 ± 0.00 ^a^	0.20 ± 0.01 ^b^	0.03 ± 0.00 ^c^	0.15 ± 0.01 ^d^
Anethol	1283	1285 [41]	1.10 ± 0.16 ^a^	0.38 ± 0.10 ^b^	0.04 ± 0.02 ^b^	4.36 ± 0.23 ^c^
Thymol	1294	1290 [41]	0.40 ± 0.04 ^a^	0.19 ± 0.04 ^b^	0.05 ± 0.00 ^c^	0.22 ± 0.01 ^b^
Carvacrol	1304	1300 [41]	0.13 ± 0.02 ^a^	0.09 ± 0.01 ^a^	-	0.21 ± 0.01 ^b^
Piperitenone	1312	1308 [2]	0.06 ± 0.00 ^a^	0.75 ± 0.07 ^b^	-	0.51 ± 0.01 ^c^
Piperitenone oxide	1333	1366 [42]	0.12 ± 0.01 ^a^	0.05 ± 0.01 ^b^	-	-
β-*E*-Caryophyllene	1418	1420 [41]	0.09 ± 0.01 ^a^	0.35 ± 0.02 ^b^	0.27 ± 0.01 ^c^	0.61 ± 0.01 ^d^
ϒ-Elemene	1434	1436 [41]	1.20 ± 0.02 ^a^	0.22 ± 0.01 ^b^	0.12 ± 0.02 ^c^	0.03 ± 0.01 ^d^
α-Humulene	1452	1453 [41]	0.09 ± 0.03 ^a^	0.09 ± 0.03 ^a^	-	0.03 ± 0.01 ^a^
Germacrene D	1479	1481 [41]	0.11 ± 0.01 ^a^	-	0.02 ± 0.00 ^b^	0.06 ± 0.01 ^c^
Bicyclogermacrene	1495	1494 [41]	0.09 ± 0.00 ^a^	-	0.03 ± 0.01 ^b^	0.10 ± 0.00 ^a^
δ-Cadinene	1528	1524 [42]	0.11 ± 0.03 ^a^	-	-	0.04 ± 0.00 ^b^
Spathulenol	1579	1578 [42]	0.29 ± 0.03 ^a^	0.10 ± 0.01 ^b^	0.08 ± 0.00 ^b^	0.19 ± 0.02 ^a^
Caryophyllene oxide	1582	1581 [41]	0.68 ± 0.04 ^a^	0.74 ± 0.14 ^b^	0.13 ± 0.00 ^c^	0.15 ± 0.01 ^c^
**Total identified**			**99.18 ± 3.13**	**96.27 ± 4.18**	**99.64 ± 0.66**	**98.45 ± 2.28**
Hydrocarbon monoterpenes			1.03± 0.29	1.91 ± 0.09	2.90 ± 0.17	2.12 ± 0.24
Oxygenated monoterpenes			95.52 ± 2.52	92.85 ± 3.61	96.09 ± 0.43	95.17 ± 1.97
Hydrocarbon sesquiterpenes			1.66± 0.20	0.67 ± 0.27	0.44 ± 0.05	0.84 ± 0.03
Oxygenated sesquiterpenes			0.97 ± 0.07	0.84± 0.15	0.21 ± 0.00	0.33 ± 0.03

Values are mean ± standard deviation of triplicate analyses. Values in the same row with different letters show statistically significant differences based on ANOVA and the Tukey test at *p* ≤ 0.05. Y1S—Year 1 Spring; Y1A—Year 1 Autumn; Y2S—Year 2 Spring; Y2A—Year 2 Autumn. * Relative quantitative data by GC-FID analysis. ** Retention index relative to C8–C22 *n*-alkanes on the Zebron ZB-5HT Inferno™. *** Retention indices reported by bibliography.

**Table 3:** Antioxidant activity (IC_50_ values, mg/mL) of *C. nepeta* essential oils.

	Antioxidant Inhibitory Activity: IC_50_ (mg/mL)		
	LPO	DPPH Radical	Reducing Power
Ascorbic acid	1.12 ± 0.06 ^a^	0.006 ± 0.001 ^a^	0.013 ± 0.001 ^a^
Y1S	1.57 ± 0.08 ^b^	27.99 ± 1.40 ^b^	25.17 ± 1.26 ^b^
Y1A	0.85 ± 0.04 ^c^	36.76 ± 1.84 ^c^	32.16 ± 1.61 ^c^
Y2S	n.d.	38.41 ± 2.51 ^c^	40.88 ± 3.81 ^d^
Y2A	n.d.	26.91 ± 1.52 ^b^	18.31 ± 1.20 ^e^

Y1S—Year 1 Spring; Y1A—Year 1 Autumn. Y2S—Year 2 Spring; Y2A—Year 2 Autumn. n.d.—not determined, due to limitations in the sample volume. IC_50_ values were calculated from three independent replicates for each EO concentration and are expressed as mean ± SD. For each assay, values with different letters in the same column indicate statistically significant differences according to ANOVA and the Tukey test at *p* ≤ 0.05.

**Table 4:** Antibacterial disc diffusion activity.

Microorganism	Inhibition Zone (mm)				
	Y1S	Y1A	Y2S	Y2A	TE (30 μg/mL)
*E. faecalis* ATCC 29212	7.5 ± 0.4 ^a,c^	**10.2 ± 0.8** ^b,e^	6.4 ± 0.2 ^c^	**t. i.** ^d^	**11.7 ± 0.9** ^e^
*E. faecalis* LFG 1001	8.3 ± 0.3 ^a^	7.8 ± 0.3 ^a^	8.1 ± 0.7 ^a^	**t. i.** ^b^	11.1 ± 0.5 ^c^
*S. aureus* ATCC 29213	**t. i.** ^a^	8.5 ± 0.4 ^b^	12.9 ± 1.1 ^c^	**26.2 ± 1.16** ^d^	**29.2 ± 0.4** ^e^
*S. aureus* LFG 1007	8.5 ± 0.5 ^a^	**20.8 ± 2.1** ^b^	12.7 ± 0.9 ^c^	**t. i.** ^d^	**37.2 ± 0.5** ^e^
*S. epidermidis* ATCC 12228	9.0 ± 0.5 ^a^	8.2 ± 0.2 ^a^	7.6 ± 0.5 ^a^	**t. i.** ^b^	9.9 ± 0.5 ^a^
*E. coli ATCC* 25922	**16.7 ± 1.8** ^a^	**18.3 ± 2.1** ^a,b^	**17.3 ± 0.9** ^a,b^	**24.0 ± 5.7** ^a,b^	**24.7 ± 0.6** ^c^
*E. coli* LFG 1003	11.7 ± 0.6 ^a^	12.3 ± 2.1 ^a^	8.4 ± 0.5 ^b,c^	11.2 ± 1.7 ^a,b^	6.7 ± 0.3 ^c^
*M. morganii* LFG 1008	**15.0 ± 0.9** ^a^	**14.5 ± 1.3** ^a^	**t. i.** ^b^	**16.2 ± 1.8** ^a^	**22.3 ± 1.8** ^c^
*P. mirabilis* LFG 1004	12.3 ± 0.8 ^a^	12.2 ± 1.3 ^a^	11.0 ± 1.7 ^a^	11.1 ± 1.5 ^a^	7.3 ± 0.7 ^b^
*P. aeruginosa* ATCC 27853	w. i.	7.8 ± 0.8 ^a^	w. i.	w. i.	**36.7 ± 1.2** ^b^
*P. aeruginosa* LFG 1002	7.0 ± 0.4 ^a^	6.0 ± 0.3 ^a^	7.2 ± 0.8 ^a^	**19.7 ± 4.7** ^b^	17.6 ± 0.5 ^b^
*S. enteritidis* LFG 1005	**16.2 ± 0.3** ^a^	9.2 ± 0.3 ^b^	8.5 ± 1.0 ^b^	**14.7 ± 1.4** ^a^	25.1 ± 0.5 ^c^
*S. typhimurium* LFG 1006	**14.5 ± 0.3** ^a^	8.2 ± 0.3 ^b^	8.0 ± 0.6 ^b^	**12.9 ± 0.8** ^c^	6.7 ± 0.3 ^d^

TE—Tetracycline hydrochloride; t. i.—total inhibition; w. i.—without inhibition. Y1S—Year 1 Spring; Y1A—Year 1 Autumn; Y2S—Year 2 Spring; Y2A—Year 2 Autumn. For each assay, values with different letters in the same column show statistically significant differences, based on ANOVA and the Tukey test at *p* ≤ 0.05.

**Table 5:** Microdilution antimicrobial activity of *C. nepeta* (MIC values).

Microorganism	MIC (μg/mL)				
	Y1S	Y1A	Y2S	Y2A	TE
*E. faecalis* ATCC 29212	**1767** ^a^	>1978	>2046	3640 ^b^	**7.8** ^c^
*E. faecalis* LFG 1001	**837** ^a^	>3956	>2046	3640 ^b^	125 ^c^
*S. aureus* ATCC 29213	**465** ^a^	>2967	>2046	3640 ^b^	**1.0** ^c^
*S. aureus* LFG 1007	**837** ^a^	**1980** ^b^	>2046	**1001** ^a^	**1.0** ^c^
*S. epidermidis* ATCC 12228	**651** ^a^	>1978	>2046	4550 ^b^	62.5 ^c^
*E. coli ATCC* 25922	**837** ^a^	**990** ^b^	**1023** ^b^	**1456** ^c^	62.5 ^d^
*E. coli* LFG 1003	**1767** ^a^	**1980** ^a,b^	2046 ^b^	2912 ^c^	500 ^d^
*M. morganii* LFG 1008	**837** ^a^	**792** ^a^	**1581** ^b^	3913 ^c^	250 ^d^
*P. mirabilis* LFG 1004	**837** ^a^	**990** ^a^	**1581** ^b^	3913 ^c^	250 ^d^
*P. aeruginosa* ATCC 27853	**837** ^a^	**990** ^a^	**1023** ^b^	3640 ^a^	125 ^c^
*P. aeruginosa* LFG 1002	**837** ^a^	**990** ^b^	**1023** ^b^	**1365** ^c^	15.6 ^d^
*S. enteritidis* LFG 1005	**837** ^a^	**792** ^a^	**1581** ^b^	2912 ^c^	**7.8** ^d^
*S. typhimurium* LFG 1006	**1767** ^a^	**1980** ^a,b^	2046 ^b^	2912 ^c^	500 ^d^

Y1S—Year 1 Spring; Y1A—Year 1 Autumn; Y2S—Year 2 Spring; Y2A—Year 2 Autumn. TE—Tetracycline hydrochloride. For each assay, values with different letters in the same column show statistically significant differences based on ANOVA and the Tukey test at *p* ≤ 0.05. Values reported as greater than the highest concentration tested (>) were excluded from the statistical analysis.

**Table 6:** Meteorological parameters and phenological growth stages of *Calamintha nepeta* during the sampling periods in Évora, Portugal.

Sample ID	Season/ Year	Mean Temp. (°C) [Anomaly]	Total Precip. (mm) [% of Normal]	Sunshine Profile/ Radiation	Dominant Abiotic Stressor/ Climatic Events	Phenological Growth Stage
Y1S	Spring (Year 1)	13.17 [−0.43]	325.9 [154%]	Coldest spring since 1993; Late frost/2nd wettest March in mainland Portugal in the last 50 years	Cold stress (Lowest May minimum temp. in 30 years).	Vegetative/pre-flowering
Y1A	Autumn (Year 1)	17.03 [+0.76]	242.2 [97%]	7th warmest autumn in 30 years; Normal precipitation.	Mechanical stress (Tornado F0/T1 event in the region).	Flowering
Y2S	Spring (Year 2)	13.11 [−0.49]	346.4 [164%]	Rainiest spring in 15 years; Record rainfall in the Lower Alentejo region.	Hydric stress (Extreme rainfall after meteorological drought).	Vegetative/pre-flowering
Y2A	Autumn (Year 2)	17.60 [+1.40]	449.5 [180%]	6th warmest & 6th rainiest since 1931; Major heatwave in Oct.	Extreme Thermal stress (Most significant Oct heatwave since 1941).	Flowering

**Note:** Climatic anomalies and percentages are based on the 1971–2000 climatological normal reported by the Portuguese Institute for Sea and Atmosphere (IPMA).

## Data Availability

The data presented in this study are available on request from the corresponding author.
